# High frequency of *CHD7* mutations in congenital hypogonadotropic hypogonadism

**DOI:** 10.1038/s41598-018-38178-y

**Published:** 2019-02-07

**Authors:** Catarina Inês Gonçalves, Filipa Marina Patriarca, José Maria Aragüés, Davide Carvalho, Fernando Fonseca, Sofia Martins, Olinda Marques, Bernardo Dias Pereira, José Martinez-de-Oliveira, Manuel Carlos Lemos

**Affiliations:** 1 CICS-UBI, Health Sciences Research Centre, University of Beira Interior, 6200-506 Covilhã, Portugal; 2 Serviço de Endocrinologia, Diabetes e Metabolismo, Hospital de Santa Maria, 1649-035 Lisboa, Portugal; 3 Serviço de Endocrinologia, Diabetes e Metabolismo, Centro Hospitalar de São João and Faculty of Medicine and Instituto de Investigação e Inovação em Saúde, University of Porto, 4200-319 Porto, Portugal; 4 Serviço de Endocrinologia, Diabetes e Metabolismo, Hospital de Curry Cabral, 1069-166 Lisboa, Portugal; 5 Unidade de Endocrinologia Pediátrica, Serviço de Pediatria, Hospital de Braga, 4710-243 Braga, Portugal; 6 Serviço de Endocrinologia, Hospital de Braga, 4710-243 Braga, Portugal; 7 Serviço de Endocrinologia e Diabetes, Hospital Garcia de Orta, 2805-267 Almada, Portugal

## Abstract

Congenital hypogonadotropic hypogonadism (CHH) is characterized by lack of normal pubertal development due to deficient gonadotropin-releasing hormone (GnRH) secretion or action, and is caused by genetic defects in several genes. Mutations in the *CHD7* gene cause CHARGE syndrome (Coloboma of the eye, Heart defects, Atresia of the choanae, Retardation of growth and development, Genital hypoplasia and Ear abnormalities), but have also been found in patients with isolated CHH. The aim of this study was to identify *CHD7* mutations in patients with CHH. Fifty Portuguese patients with CHH were screened for mutations in the *CHD7* gene by DNA sequencing. Eight (16%) patients had *CHD7* rare sequence variants that consisted of six missense (p.Gly388Glu, p.His903Pro, p.Thr1082Ile, p.Val1452Leu, p.Asp1854Gly, and p.Arg2065His) and two synonymous (p.Ser559Ser, and p.Ala2785Ala) mutations. Five of these mutations have never been reported before. Three *CHD7* mutations occurred in patients that had mutations in additional CHH-genes. This study uncovered novel genetic variants that expand the known spectrum of mutations associated with CHH. The frequency of *CHD7* mutations in this cohort was higher than that of other major CHH-genes and confirms the importance of including *CHD7* in the genetic testing of CHH, even in the absence of additional CHARGE features.

## Introduction

Congenital hypogonadotropic hypogonadism (CHH) is characterized by partial or complete lack of pubertal development, secondary to deficient gonadotropin-releasing hormone (GnRH) induced gonadotropin secretion^1^. The diagnosis is based on the existence of low levels of sex hormones associated with low or inappropriately normal levels of luteinizing hormone (LH) and follicle-stimulating hormone (FSH). CHH may occur associated with anosmia, a condition referred to as Kallmann syndrome (KS), or may occur without associated olfactory abnormalities, referred to as normosmic CHH (nCHH)^1^.

Monogenic or oligogenic defects are found in about 50% of patients with CHH, in genes that regulate the embryonic development or migration of GnRH neurons, or the synthesis, secretion or action of GnRH^2^. Mutations in the *ANOS1* (*KAL1*) (MIM 300836), *FGFR1* (*KAL2*) (MIM 136350) and *GNRHR* (MIM 138850) genes have been the most frequently reported, but there are now over 30 genes that have been associated with CHH. However, most of these genes are rarely involved in CHH and even the most commonly implicated genes usually account for less than 10% of cases^2^.

CHARGE syndrome is a rare disorder characterized by a variable combination of congenital anomalies that include Coloboma of the eye, Heart defects, Atresia of the choanae, Retardation of growth and development, Genital hypoplasia and Ear abnormalities^3^. Heterozygous loss-of-function mutations in the chromodomain helicase DNA-binding protein 7 (*CHD7*) gene (MIM 608892) are the major cause of CHARGE syndrome^4^. Hypogonadotropic hypogonadism and olfactory defects, which are the hallmarks of KS, are commonly observed in CHARGE syndrome^5^ and *CHD7* mutations have been identified in patients with isolated CHH (i.e. without additional CHARGE features)^6–11^.

The aim of this study was to determine the frequency of *CHD7* mutations in a cohort of patients with isolated CHH.

## Material and Methods

### Subjects

The study comprised 50 unrelated Portuguese patients with CHH (42 men and 8 women, mean age at diagnosis 21.4 years, range 14–45 years), 22 with KS and 28 with nCHH, recruited by Portuguese clinical endocrine centers. Inclusion criteria were patients with low or inappropriately normal serum FSH, LH and sex steroid levels, failure to enter spontaneous puberty by the age of 18 years or with medically induced puberty below this age, and absence of other pituitary hormone deficiencies. Olfactory function was assessed either by olfaction testing or self-reported by the patients, depending on the clinical center. None of the patients had diagnostic criteria for CHARGE syndrome^12^. In mutation-positive patients, additional family members were studied, when available. The control population consisted of >200 Portuguese unrelated healthy volunteers who were recruited among blood donors. The study was approved by the local research ethics committee (Faculty of Health Sciences, University of Beira Interior, Ref: CE-FCS-2012-012). Written informed consent was obtained from all subjects and all methods were performed in accordance with the relevant guidelines and regulations.

### Genetic studies

Genomic deoxyribonucleic acid (DNA) was extracted from peripheral blood leucocytes using previously described methods^13^. Patients had already been screened for mutations in the *ANOS1*, *FGFR1* and *GNRHR* genes, resulting in the discovery of three mutations in *ANOS1*^14^, six mutations in *FGFR1*^15^ and six mutations in *GNRHR*^16^. All 50 patients were subsequently screened for mutations in the *CHD7* gene by polymerase chain reaction (PCR) amplification of the coding exons and exon-intron boundaries, and bi-directional sequencing using CEQ DTCS sequencing kit (Beckman Coulter, Fullerton, CA, USA) and an automated capillary DNA sequencer (GenomeLab TM GeXP, Genetic Analysis System, Beckman Coulter). Primer sequences for *CHD7* were previously described by Song *et al*.^17^, except for the primer sequence of exons 2.4 and 3, which were designed using Primer 3 Plus^18^. Genomic sequence variants identified in patients were searched in the Exome Aggregation Consortium (ExAC) population variant database^19^, to assess their frequency in the general population. Variants found to be absent in the ExAC database or with frequencies <0.1% were further screened in a panel of at least 200 healthy Portuguese volunteers (400 alleles), using allele-specific PCR or sequence-specific restriction enzymes, to exclude the possibility that they represented common polymorphisms in the Portuguese population. Variants were considered to be pathogenic when they were simultaneously found to have an ExAC population frequency <0.1%, to be absent in the Portuguese control population, and to have a deleterious effect predicted by at least one of four bioinformatic programs (SIFT^20^, PolyPhen-2^21^, Mutation Taster^22^ or Human Splicing Finder^23^). Sequence variant nomenclature followed standard guidelines^24^ and was based on the cDNA reference sequence for the *CHD7* gene (GenBank accession NM_017780.3). Patients with pathogenic variants in *CHD7* were further screened for mutations in additional genes related to the hypothalamic–pituitary–gonadal axis (*PROKR2*, *PROK2*, *FGF8*, *GNRH1*, *KISS1R*, *TAC3* and *TACR3*), to identify possible cases of oligogenicity (primer sequences and PCR conditions are available upon request).

## Results

**Figure 1:**
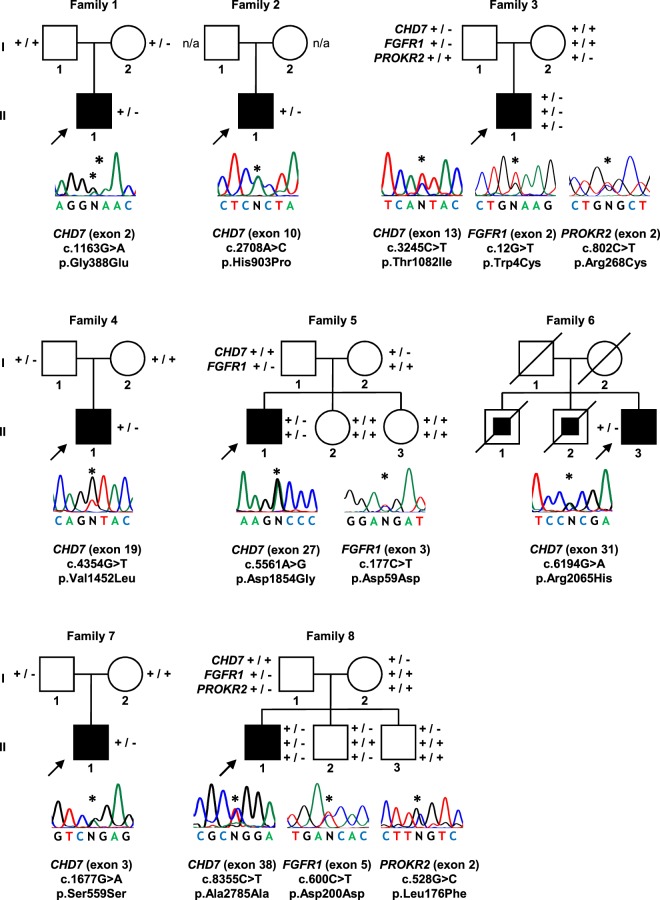
*CHD7* mutations identified in patients with CHH. Arrows represent index cases, filled symbols represent affected individuals, open symbols represent unaffected individuals, squares denote men, circles denote women, oblique lines through symbols represent deceased individuals. Filled squares within squares represent individuals reported to have heart defects associated with polydactyly. Patients from families 3, 5 and 8 had mutations in additional genes, thus representing cases of oligogenicity. Genotypes for additional family members, when available, are presented beside each individual (+, wild-type allele; −, mutated allele; n/a, not available for genetic studies). The position of each heterozygous mutation on the DNA sequence is indicated by an asterisk.

Sequence analysis of the entire coding region and exon–intron boundaries of *CHD7* revealed 13 heterozygous variants that had frequencies <0.1% in the ExAC population database. Five of these variants were detected in normal Portuguese controls (p.Thr689Thr, 2 of 408 alleles; p.Lys729Glu, 1 of 436 alleles; c.2613 + 4 C > T, 1 of 454 alleles; p.Gly1479Gly, 1 of 398 alleles; p.Pro2072Pro, 1 of 450 alleles) and were excluded from further analysis. The remaining eight variants were found to be absent in the Portuguese control population and were predicted to cause changes in protein function, by at least one bioinformatic prediction program (Table 1). Thus, 16% (8 out of 50) of patients with CHH were considered to have pathogenic variants in *CHD7*. Mutations occurred in both KS (4/22, 18%) and nCHH (4/28, 14%). Mutations consisted of six missense (c.1163 G > A, p.Gly388Glu; c.2708 A > C, p.His903Pro; c.3245 C > T, p.Thr1082Ile; c.4354 G > T, p.Val1452Leu; c.5561 A > G, p.Asp1854Gly; c.6194 G > A, p.Arg2065His) and two synonymous (c.1677G > A, p.Ser559Ser; c.8355 C > T, p.Ala2785Ala) variants (Fig. 1). Conservation analysis revealed that, with the exception of p.Gly388Glu, all missense variants occurred at amino acids that were highly conserved across species (Supplemental Table 1).

**Table 1:** Rare sequence variants predicted to be pathogenic by at least one computational program.

*CHD7* variant	Population allele frequency (ExAC/Portuguese)^(a)^	Computational programs that support a pathogenic effect^(b)^	Additional genetic variants
**Missense**			
c.1163 G > A; p.Gly388Glu	0.0008%/0%	SIFT, PP2, MT	
c.2708 A > C; p.His903Pro	0%/0%	SIFT, PP2, MT	
c.3245 C > T; p.Thr1082Ile	0%/0%	SIFT, PP2, MT	*FGFR1* c.12 G > T and *PROKR2* c.802 C > T^(c)^
c.4354 G > T; p.Val1452Leu	0%/0%	MT	
c.5561 A > G; p.Asp1854Gly	0%/0%	SIFT, PP2, MT	*FGFR1* c.177 C > T
c.6194 G > A; p.Arg2065His	0%/0%	SIFT, PP2, MT	
**Synonymous**			
c.1677G > A; p.Ser559Ser	0.0036%/0%	MT, HSF	
c.8355 C > T; p.Ala2785Ala	0.0074%/0%	MT, HSF	*FGFR1* c.600 C > T and *PROKR2* c.528 G > C

^(a)^ExAC Exome Aggregation Consortium frequency/Portuguese control population. ^(b)^SIFT, Sorting Tolerant From Intolerant; PP2, PolyPhen-2; MT, Mutation Taster; HSF, Human Splicing Finder. ^(c)^Patient previously reported by Gonçalves *et al*.^15^.

The p.Ser559Ser, p.Arg2065His and p.Ala2785Ala variants, have been previously identified in patients with CHARGE syndrome^25^. The remaining five variants have not yet been reported in patients with either CHH or CHARGE syndrome.

In six of the eight families, it was possible to determine the parental origin of the *CHD7* mutation and in all six cases the mutation was transmitted by a parent who did not have CHARGE symptoms or a history of delayed puberty (Fig. 1). In three of these cases, patients presented mutations in additional CHH genes, namely one digenic mutation (*CHD7*/*FGFR1*) and two trigenic mutations (*CHD7*/*FGFR1*/*PROKR2*), thus representing cases of oligogenic inheritance (Fig. 1).

All *CHD7* variants were submitted to the *CHD7* mutation database at www.chd7.org.

The clinical characteristics of patients with identified *CHD7* pathogenic variants are summarized in Table 2.

**Table 2:** Clinical characteristics of patients with *CHD7* rare sequence variants.

Patient	Sex	Age of diagnosis (yrs)	Clinical presentation	Olfaction	Associated features	Basal hormone levels (laboratory normal reference range)	Brain MRI/CT	Sequence variants (heterozygous)
1	M	14	Micropenis, cryptorchidism and delayed puberty	Anosmia	—	FSH 0.29 mIU/mL (1.1–13.6); LH < 0.07 mIU/mL (1.1–8.8); total T 0.34 ng/mL (2.8–11.1)	Pituitary hypoplasia	*CHD7* c.1163 G > A, p.(Gly388Glu)
2	M	14	Micropenis and delayed puberty	Normal	—	n/a	Normal	*CHD7* c.2708 A > C, p.(His903Pro)
3	M	19	Arrested puberty	Normal	Osteoporosis and osteopenia (lumbar spine Z-score −2.6; femoral neck Z-score −2.1).	FSH 2.6 mIU/mL (1.0–12.0); LH 2.0 mIU/mL (1.0–7.0); total T 0.32 ng/mL (2.60–10.00)	Normal	*CHD7* c.3245 C > T, p.(Thr1082Ile) and *FGFR1* c.12 G > T, p.(Trp4Cys) and *PROKR2* c.802 C > T, p.(Arg268Cys)
4	M	21	Delayed puberty	Anosmia	Reversal of hypogonadism after testosterone replacement therapy (age 33 yrs).	FSH 1.7 mIU/mL (1.5–12.0); LH 0.9 mIU/mL (1.0–8.5); total T 0.7 ng/mL (2.6–16.0)	Normal	*CHD7* c.4354 G > T, p.(Val1452Leu)
5	M	17	Micropenis, cryptorchidism and delayed puberty	Anosmia	Mild hearing deficit.	FSH < 0.3 mIU/mL (1.4–10.0); LH < 0.07 mIU/mL (1.5–9.3); total T < 0.2 ng/mL (2.4–8.3)	Normal	*CHD7* c.5561 A > G, p.(Asp1854Gly) and *FGFR1* c.177 C > T, p.(Asp59Asp)
6	M	16	Cryptorchidism and delayed puberty	Anosmia	Recurrent pyelonephritis. Renal transplant due to chronic renal failure (age 43 yrs).	FSH 3.4 mIU/mL (5.0–25.0); LH 1.9 mIU/mL (5.0–30.0); total T 0.87 ng/mL (2.50–7.50)	Normal	*CHD7* c.6194 G > A, p.(Arg2065His)
7	M	15	Micropenis and delayed puberty	Normal	Psicomotor developmental delay. Strabismus. Surgery for deviated nasal septum and hypertrophy. Reversal of hypogonadism after testosterone replacement therapy (age 18 yrs).	FSH 0.71 mIU/mL (0.8–8.2); LH < 0.07 mIU/mL (0.7–7.2); total T 0.22 ng/mL (2.20–8.00)	Normal	*CHD7* c.1677G > A, p.(Ser559Ser)
8	M	17	Delayed puberty	Normal	—	FSH 0.3 mIU/mL (0.8–8.2); LH < 0.1 mIU/mL (0.7–7.2); total T 0.05 ng/mL (2.20–8.00)	Pituitary hypoplasia	*CHD7* c.8355 C > T, p.(Ala2785Ala) and *FGFR1* c.600 C > T, p.(Asp200Asp) and *PROKR2* c.528 G > C, p.(Leu176Phe)

M, male; yrs, years; FSH, follicle stimulating hormone; LH, luteinizing hormone; T, testosterone; MRI, magnetic resonance imaging; CT, computerized tomography; n/a, not available. Hormone levels are only presented for gonadotrophins and testosterone, all other pituitary hormone measurements were normal.

## Discussion

The overall prevalence of *CHD7* mutations in this cohort of Portuguese patients with CHH was 16% (8 out of 50), with a similar distribution between the KS and nCHH forms. This is a high prevalence when compared to the contribution of other genes that have been historically considered as priorities in the genetic study of CHH, namely the *ANOS1* (*KAL1*), *FGFR1*, and *GNRHR* genes^26^. Indeed, previous studies in the Portuguese population have shown that the *ANOS1* (*KAL1*), *FGFR1*, and *GNRHR* genes are mutated in only 7.1%, 12.0%, and 12.5% of cases, respectively^14–16^. Studies in other populations have also shown an important contribution of *CHD7* mutations in the aetiology of CHH, with frequencies ranging from 5.2% to 19.0%, of cases^6–11^. Thus, the *CHD7* gene is becoming increasingly recognised as one of the most commonly mutated genes in CHH.

Although *CHD7* mutations can cause both CHARGE syndrome and isolated CHH, it is likely that this variable phenotypic expression is related to the severity of the *CHD7* mutations, as mutations in CHARGE syndrome are typically highly deleterious protein-truncating mutations, whereas *CHD7* mutations in isolated CHH are typically missense^8^. Accordingly, in our CHH patients, all identified *CHD7* mutations were either missense (n = 6) or synonymous (n = 2).

The eight *CHD7* mutations identified in this study were found to be absent or very rare (<0.1%) in the ExAC population database^19^, absent in Portuguese controls, and predicted to be damaging by structure- and sequence-based bioinformatics programs^20–23^. Three of these mutations (one missense and two synonymous) have already been reported in patients with CHARGE syndrome^25^, and the remaining five missense mutations have not yet been reported in patients with either CHH or CHARGE syndrome. The missense mutations occurred at highly conserved amino acids. Furthermore, the p.His903Pro, p.Thr1082Ile and p.Val1452Leu mutations are located in chromodomain 2, the SNF2 domain and the helicase domain, respectively, which play important roles in the chromatin remodelling activity of the CHD7 protein^6^. The remaining missense mutations occurred outside these catalytic domains but were also predicted to affect protein function. The mechanisms by which the synonymous mutations exert their effect were not possible to determine. However, synonymous mutations - sometimes called ‘silent’ mutations - are widely acknowledged to be able to cause changes in protein expression, conformation and function, and have been implicated in several diseases^27^. It should be noted however that according to more stringent classification criteria recommended by the American College of Medical Genetics and Genomics (ACMG)^28^, only the p.Thr1082Ile variant is considered “likely pathogenic” and the remaining are considered “variants of uncertain significance”.

Three patients with *CHD7* mutations presented additional defects in known CHH-genes. Thus, these represent cases of oligogenic inheritance, which is a frequent genetic finding in CHH^29^. Oligogenicity might explain some cases of incomplete penetrance, where carriers of a *CHD7* mutation only express CHH in the presence of other mutated genes.

Our patients with *CHD7* mutations did not undergo detailed radiological investigations to detect hypoplasia/agenesis of semicircular canals or of olfactory bulbs and tracts, which are currently major criteria for CHARGE syndrome^30,31^. Thus, we cannot exclude that patients have light forms of CHARGE syndrome. Other studies have shown that a subset of patients with apparently isolated CHH, in whom a *CHD7* defect was demonstrated, were subsequently found to exhibit multiple CHARGE features and reclassified as having CHARGE syndrome^32–34^. Interestingly, three of our patients with *CHD7* mutations had minor CHARGE features^30,31^, namely hearing deficit, renal anomalies and intellectual disability, respectively, but insufficient for a clinical diagnosis of CHARGE syndrome. It remains to be determined if these were coincidental findings or the result of the *CHD7* mutations.

Our results should be viewed with caution as *in vitro* and *in vivo* functional tests are not readily available for *CHD7*^35^, and therefore it was not possible to confirm the damaging effect of the observed *CHD7* variants. In addition, a limited number of CHH-genes was analysed by Sanger sequencing and it remains to be determined if a more comprehensive genetic analysis (e.g. through whole exome sequencing) would uncover additional cases of oligogenicity and explain the incomplete penetrance observed in some families. Finally, CHH is clinically heterogeneous and sometimes overlaps with constitutional delay of growth and puberty (CDGP)^36^. Our study included patients with a wide range of ages and we cannot exclude the possibility that some of the younger patients, who underwent medically induced puberty, may represent cases of CDGP or mild forms of CHH that would have eventually developed spontaneous late puberty. Therefore, our results may not be directly comparable with those of other studies that used different inclusion criteria.

In conclusion, our study identified a high frequency of *CHD7* mutations in patients with CHH and uncovered novel genetic variants that expand the known spectrum of mutations associated with CHH.

## Supplementary information

Supplemental Table S1
