# Supplementary material for: High frequency of CHD7 mutations in congenital hypogonadotropic hypogonadism
Source: Sci Rep. 2019 Feb 7;9:1597. doi: 10.1038/s41598-018-38178-y (PMC6367338; doi:10.1038/s41598-018-38178-y)
Supplement: Supplementary file 1 — Supplemental Table S1 [file 41598_2018_38178_MOESM1_ESM.pdf]

High frequency of CHD7 mutations in congenital hypogonadotropic hypogonadism

Catarina Inês Gonçalves, Filipa Marina Patriarca, José Maria Aragüés, Davide Carvalho, Fernando Fonseca, Sofia Martins, Olinda Marques, Bernardo Dias Pereira, José Martinez-de-Oliveira, Manuel Carlos Lemos.

Supplemental Table S1. Evolutionary conservation of CHD7 mutated amino acids (bold) across different species.

	G388E		H903P		T1082I	
Species	Codon	Alignment	Codon	Alignment	Codon	Alignment
Human	388	P S Q P Q **G** T Y A S P	903	G E P V T **H** Y L V K W	1082	F H A I I **T** T F E M I
Mutated variant	388	P S Q P Q **E** T Y A S P	903	G E P V T **P** Y L V K W	1082	F H A I I **I** T F E M I
P.troglodytes	388	P S Q P Q **E** T Y A S P	903	V T **H** Y L V K W	1082	I **T** T F E M I
M.mulatta	388	P S Q P Q **E** T Y A S P	903	V T **H** Y L V K W	1082	I **T** T F E M I
F.catus						
M.musculus	382	P S Q P Q **E** T Y A S P	893	P V I **H** Y L V K W	1072	I I **T** T F E M I
G.gallus			141	P V T **H** Y L V K W	320	F H A I I **T** T F E M I
T.rubripes						
D.rerio	402	A Q P Q **G** S Y S S P	947	G E L V T **L** Y L V K W	1126	F H A V I **T** T F E M I
D.melanogaster			1962	G E T T K **H** Y L V K W	2143	F N V L I **T** T F E M I
C.elegans	544	P H Q H Q **Q** P A S V P	1135	G Q E F V **-** - L I K W	1311	I D A L I **T** T F E T V
X.tropicalis	323	P S Q P Q **G** T Y A S P	830	G E P V C **H** Y L V K W	1009	F H A I I **T** T F E M I

	V1452L		D1854G		R2065H	
Species	Codon	Alignment	Codon	Alignment	Codon	Alignment
Human	1452	N A T N G **V** Q Q L S K	1854	D R E D E **D** P E Y K P	2065	L L R K I **R** E Q V L H
Mutated variant	1452	N A T N G **L** Q Q L S K	1854	D R E D E **G** P E Y K P	2065	L L R K I **H** E Q V L H
P.troglodytes	1452	N A T N G **V** Q Q L S K	1854	D R E D E **D** P E Y K P	2065	L L R K I **R** E Q V L H
M.mulatta	1452	N A T N G **V** Q Q L S K	1856	D R E D E **D** P E	2067	L L R K I **R** E Q V L H
F.catus						
M.musculus	1442	N A T N G **V** Q Q L S K	1844	D R E D E **D** P E Y K P	2055	L L R K I **R** E Q V L H
G.gallus	690	N A T N G **V** Q Q L S K	1092	D R E D E **D** P E Y K P	1302	L L R K I **R** E Q V L H
T.rubripes						
D.rerio	1504	N A A N G **V** Q Q L S K	1906	E E E **D** P E Y K P	2149	L L R R I **R** E Q V L P
D.melanogaster	2512	S K D G N **V** K Q L S K	2991	K H D D G **D** E V D D D		
C.elegans	1683	A E G T A **-** - - L S K				
X.tropicalis	1382	N A T N G **V** Q Q L S K	1784	E **D** P E Y K P	1993	L L R K I **R** E Q V L N
